# Synthesis of Ce-doped Mn_3_Gd_7−*x*_Ce_*x*_(SiO_4_)_6_O_1.5_ for the enhanced catalytic ozonation of tetracycline

**DOI:** 10.1038/s41598-019-55230-7

**Published:** 2019-12-10

**Authors:** Jie Fu, Ning Liu, Lefu Mei, Libing Liao, Dina Deyneko, Jiayang Wang, Yaning Bai, Guocheng Lv

**Affiliations:** 1 Beijing Key Laboratory of Materials Utilization of Nonmetallic Minerals and Solid Wastes, National Laboratory of Mineral Materials, School of Materials Sciences and Technology, China University of Geosciences, Beijing, 100083 China; 2 Chemistry Department, Lomonosov Moscow State University, 119991 Moscow, Russia

**Keywords:** Environmental chemistry, Materials for energy and catalysis, Structural materials

## Abstract

A novel cerium doped compounds Mn_3_Gd_7–*x*_Ce_*x*_(SiO_4_)_6_O_1.5_ with an apatite structure was found and used to achieve high-efficiency degradation of tetracycline in aqueous solution. The catalysts were characterized by XRD, XPS, EDS and other techniques. The characteristic results indicated that the catalytic activity of the compound was improved due to the introduction of Ce in the structure, because Ce^3+^ which was stably present in the apatite structure can serve as an active site for the reaction, and in addition, there was a high presence between Ce^4+^ and Ce^3+^ on the surface of the catalyst. The redox potential and high oxygen storage capacity were also beneficial for the catalytic reaction. The results of free radical capture indicated that both superoxide radicals and hydroxyl radicals participated in the catalytic oxidation process and played an important role in the reaction. The decomposition of tetracycline followed the pseudo second-order reaction kinetics. In addition, the catalyst exhibited long-term stability and low metal leaching during the reaction, which indicated that the novel cerium-doped apatite structure material could be a promising wastewater treatment material.

## Introduction

Antibiotics are extensively used in the treatment of human diseases and in livestock and aquaculture, due to their broad-spectrum and extremely strong antibacterial activity^1^. In recent years, with the extensive use of antibiotics in human and veterinary medicine, trace antibiotics have been detected the in drinking water, surface water, groundwater, soil, and aquatic organisms^2^. Due to their low metabolism rate, some antibiotics remain in the natural environment for a long time, which not only damage the balance of the ecological environment, but also causes detrimental to humans, due to their enrichment in food chains or pollution of drinking water^4,5^. Therefore, alongside the plans of reasonable use of antibiotics in medicines, it is important also to develop effective treatment technologies to remove antibiotics from polluted water or soil^3^.

Tetracycline (TC) exists in trace levels in natural waters and is difficult to decompose, and the toxicity of its primary decomposition products is comparable to or even higher than that of the parent compound^4^. Therefore, traditional techniques including adsorption separation^5^ and biodegradation^6^ have been investigated to remove antibiotics and other antimicrobials from water. However, the adsorption method does not destroy the structure of tetracycline and cannot achieve the effect of thorough removal. On the other hand, TC inhibits metabolism during microbial degradation. Thus, rapid and efficient treatment processes must be developed for TC degradation^1^.

Advanced oxidation processes (AOPs) is based on highly potent chemical species^7^. It has the characteristics of high efficiency^8^, bottom measurement, the absence of secondary pollution^9^ and short residence time for the treatment of refractory organic matter in water and sewage. Compared with the traditional method of wastewater treatment, it has obvious advantages such as strong oxidizing ability, non-selective simple reaction conditions, and no requirements for high temperature and pressure. It can be used not only for advanced treatment of sewage, but also in combination with other treatment technologies^10^. In recent years, heterogeneously catalyzed ozone oxidation technology has received extensive attention in the large-scale water treatment^11^. Meanwhile, in order to achieve more efficient processing efficiency, catalysts for heterogeneous catalytic ozonation technology include transition metal oxide such as manganese dioxide, iron oxide, copper oxide, rare earth oxides, such as cerium oxide and their composites, have been reported as solid supports for the catalytic degradation. However, regardless if they are single metal oxide or compounds, there will be a certain amount of metal dissolution^12,13^, causing catalyst deactivation and secondary pollution, wasting of resources. Therefore, increasingly researches are focused on the way to low the amount of metal dissolution, in which introducing catalytically active metal ions into the crystal lattice is a good approach. In the context of lowing the active ion dissolution. doping of the crystal lattice with active metal ions, zhu *et al*.^14^ studied the A-position of the perovskite compound has 12-fold coordinated positions, and the high coordination number of the active ions can effectively improve the stability of the active metal in the compound, otherwise, Wang *et al*.^15^ also reported relationship between leaching ratio and doping content. And further, by using the active metal doping into the crystal lattice can also better disperse the active elements into the crystal lattice to better improve the catalytic performance of the sample. S.I. Suárez-Váquez *et al*. also reported the phenomenon^16^, the addition of Mn resulted in the incorporation of Mn^4+^ in Ti^4+^ sites present into the structure of the perovskite. This incorporation also enhances the relation O_ads_/O_latt_ and the catalytic properties. Finally, the catalyst doped by Mn presented the highest catalytic activity^17^. In addition, CNTs attached by means of CH-π, π-π stacking and Van der Waals forces could make them disperse in liquid media and leave the polyaromatic pattern unaltered, and reduce catalyst deactivation.

The compounds with an apatite-type structure A_10_[MO_4_]_6_O_2_ have great flexibility in their crystal lattice to accommodate a big number of substitutions^18^. The cations in a position can be substituted by foreign cations having different oxidation states or radii, thus increasing the number of the cation vacancies in the structure. Meanwhile, [PO_4_]^3−^ ions can also be substituted by [SiO4]^4−^, [GeO_4_]^4−^ anion groups under different conditions. Obviously, the component adjustment will bring a bit of active sites, to improve catalytic effect. So, it is a kind of potential candidate for catalyst designing technology.

Among the different reported catalysts, transition metal ions such as Mn(II), has demonstrated high efficiencies in catalytic ozonation for homogeneous degradation of various organic pollutants^19^. Manganese ion with the lowest state has a significant advantages as a redox medium for the removal of organic pollutants^20^. Besides, via the introduction of MnO_*x*_, large amount of surface hydroxyl groups are generated on the surface of catalyst, it play a key role in degradation adsorption and ·OH initiation, Higher multivalent MnO_*x*_ (Mn(III)/Mn(IV)) enhances electron transfer, which also benefits degradants removal^17^. To improve the catalytic activity, cerium (Ce) with a high oxygen storage capacity has been commonly used^21^. As such, a redox cycle between the +3 and +4 states can be manipulated to create efficient catalysts process^3^. Interestingly, the doped Ce cations can enter the apatite-type lattice with equivalent substitution, and maintain the stability of the structure. Inclusion of foreign metals in the structure could lead to increases in defects inside the catalyst surface thereby creating more number of active sites^22,23^.

In this work, we prepared a kind of Ce-doped apatite-type compounds Mn_3_Gd_7−*x*_Ce_*x*_(SiO_4_)_6_O_1.5_ using traditional high temperature solid phase method and applied it in the catalytic ozonation of TC. The results showed that strong interactions between Ce atoms in apatite-type structure were established and promoted the regeneration of the catalyst and extended its lifecycle. Also, it was shown that the new composite had a high removal efficiency of TC in ozone catalytic degradation. In addition, the pathways and mechanism of TC degradation were proposed, and had a good stability performance after the reaction.

## Experimental and Methods

A traditional high temperature solid-state reaction was used to prepare the Mn_3_Gd_7−*x*_Ce_*x*_(SiO_4_)_6_O_1.5_ compounds. The raw materials including MnCO_3_ (Aldrich, 99.9%), SiO_2_ (Aldrich, 99.9%) and Gd_2_O_3_ (Aldrich, 99.99%) and CeO_2_ (Aldrich, 99.99%) used for the syntheses of Mn_3_Gd_7−*x*_Ce_*x*_(SiO_4_)_6_O_1.5_ were purchased from the the Sinopharm Chemical Reagent Co., Ltd. Firstly, calculating the amount of each raw material required according to the stoichiometric ratio, after weighting and thoroughly mixing in the agate mortar, the mixtures were placed into little corundum crucibles, and covered with activated carbon to prevent oxidation. Finally, the samples were sintered at 1200 °C for 4 h in muffle furnace to produce the final products.

The crystal structures of the synthesized samples were examined by the X-ray powder diffractometer (XRD; D8 Advance diffractometer, Germany) with CuKα radiation (λ = 1.5418 Å) from 10° to 70° (2θ). The valence of manganese and cerium in the structure of Mn_3_Gd_7−*x*_Ce_*x*_(SiO_4_)_6_O_2_ was confirmed by X-ray Photoelectron Spectroscopy (XPS, Thermo Scientific) with monochromatic AlKa irradiation (150 W). The binding energy (BE) scale was calibrated in reference^23^ to the energy of the adventitious carbon (C 1 s) core level assigned at 284.6 eV. The visible spectra of as-prepared samples were performed on ultraviolet-visible spectroscopy (Beijing North Temple Instrument Technology Co., Ltd.) to collect the wavelength range from 500 to 700 nm at an interval of 1 nm. The molecular weight of the obtained samples was identified by methods of HPLC-mass spectrometer (LC-MS. Thermo Scientific) with high resolution search (Thermo Scientific)^24^. Catalytic ozonation processes were carried out in a semi-batch reactor containing 0.4 g/L of the target pollutant. The experimental device is composed of an ozone generating device, a vapor-liquid reaction device, a stirring device and an exhausting gas treating device. Ozone was produced by an ozone generator and passed into the reaction unit at 500 cm^3^•min^−1^, and the concentration of ozone is 40 mg/L. The reaction vessel was a 500 mL three-necked flask, the temperature in the vessel was maintained at 20 °C, and the agitation rate was set at 500 rpm•min^−1^. The exhaust gas treatment unit is a 500 mL 25 g/L Na_2_S_2_O_3_ solution. At certain time intervals, water samples were withdrawn from the reactor with a syringe and collected to measure the TC concentration. To test the stability and recyclability of Mn_3_Gd_7−*x*_Ce_*x*_(SiO_4_)_6_O_1.5_, the catalyst was filtered, centrifuged at 10,000 rpm, dried at 80 °C, and used again in another cycles.

## Results and Discussion

### Characterization of the materials

**Figure 1:**
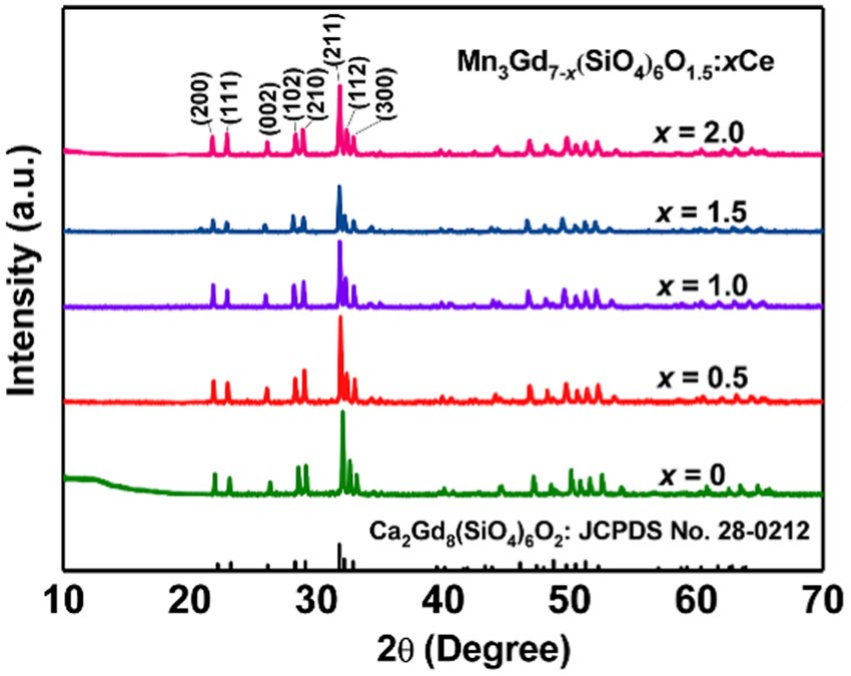
XRD patterns of Mn_3_Gd_7−*x*_(SiO_4_)_6_O_1.5_: *x*Ce particles, and the standard data for Gd_2_Gd_8_(SiO_4_)_6_O_2_ (JCPDS card No. 28-0212) is shown as a comparison.

**Figure 2:**
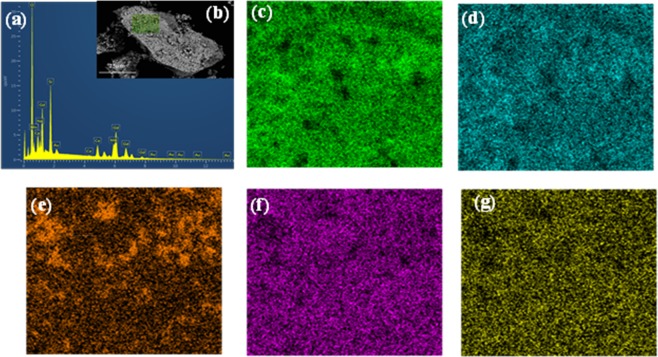
Catalyst on the degradation of TC, cycle stability test: (**a**) five times cycles, (**b**) XRD test of catalyst after every times using; leaching amount test: (**c**) leaching amount of Ce before and after recycle.

Figure 1 shows the XRD patterns of the structure of Mn_3_Gd_7–*x*_Ce_*x*_(SiO_4_)_6_O_1.5_ solid solution along the *c* axis. Two cationic sites exist in the structure: 9-fold coordinated *4f* sites with C_3_ point symmetry and 7-fold coordinated 6 h sites with *C*_s_ point symmetry^18^. To verify the phase purity and structure, Mn_3_Gd_7–*x*_Ce_*x*_(SiO_4_)_6_O_1.5_ with different amount cerium doping were characterized by XRD. The pure phases of the solid solution were obtain, and no peaks of other impurity phases were found. All the characterized peaks are in a good agreement with the standard Ca_2_Gd_8_(SiO_4_)_6_O_2_ (JCPDS No.28-0212). Although the substitution amount of Ce element is increased, the structure of the compound remains isostructure with Ca_2_Gd_8_(SiO_4_)_6_O_2_. Crucially, the proportion of positive ions was greater than that of negative ions in the sample, which provides direct evidence of the existence of vacancies^25^. Moreover, XRD patterns of the Mn_3_Gd_7−*x*_Ce_*x*_(SiO_4_)_6_O_1.5_ samples after every cycles of TC degradation were shown in Fig. 2(b), and will be discussed in the following sections.

**Figure 3:**
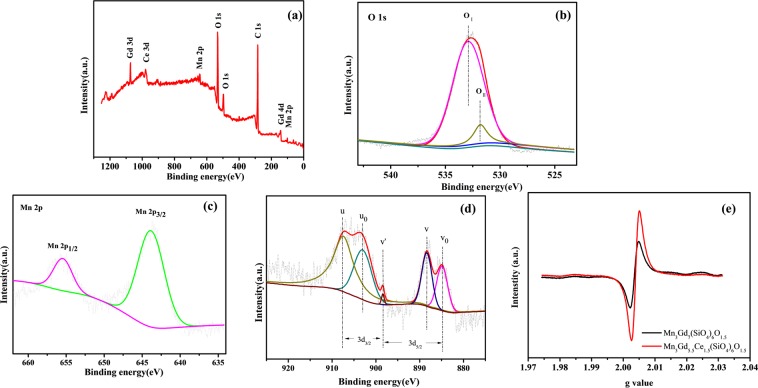
(**a**) EDS spectrum, (**b**) SEM image, (**c**–**g**)Elemental analysis of Mn_3_Gd_5.5_Ce_1.5_(SiO_4_)_6_O_1.5_ particles about O, Si, Mn, Gd, and Ce respectively.

The Energy dispersive spectroscopy (EDS) were carried out for the chemical composition of the compounds prepared in the study. The result of a scanning electron microscope (SEM) image of a Mn_3_Gd_5.5_Ce_1.5_(SiO_4_)_6_O_1.5_ sample was shown in Fig. 3(a). The particles of the sample are not uniform and the particles are relatively large due to agglomeration during heating. Figure 3(b) shows an EDS elemental analysis of surface points in a rectangular region, the proportion of positive-charged ions was greater than that of negative ions in the sample, which provides direct evidence of the existence of vacancies^25^. In addition, Fig. 3(c–g) shows the elemental distributions of O, Si, Mn, Gd, and Ce in Mn_3_Gd_5.5_Ce_1.5_(SiO_4_)_6_O_1.5_ particles. The Mn element is less homogeneous than the remaining elements, we think this might be due to the morphology of the samples and an increased amount near the surface of the particles.

**Figure 4:**
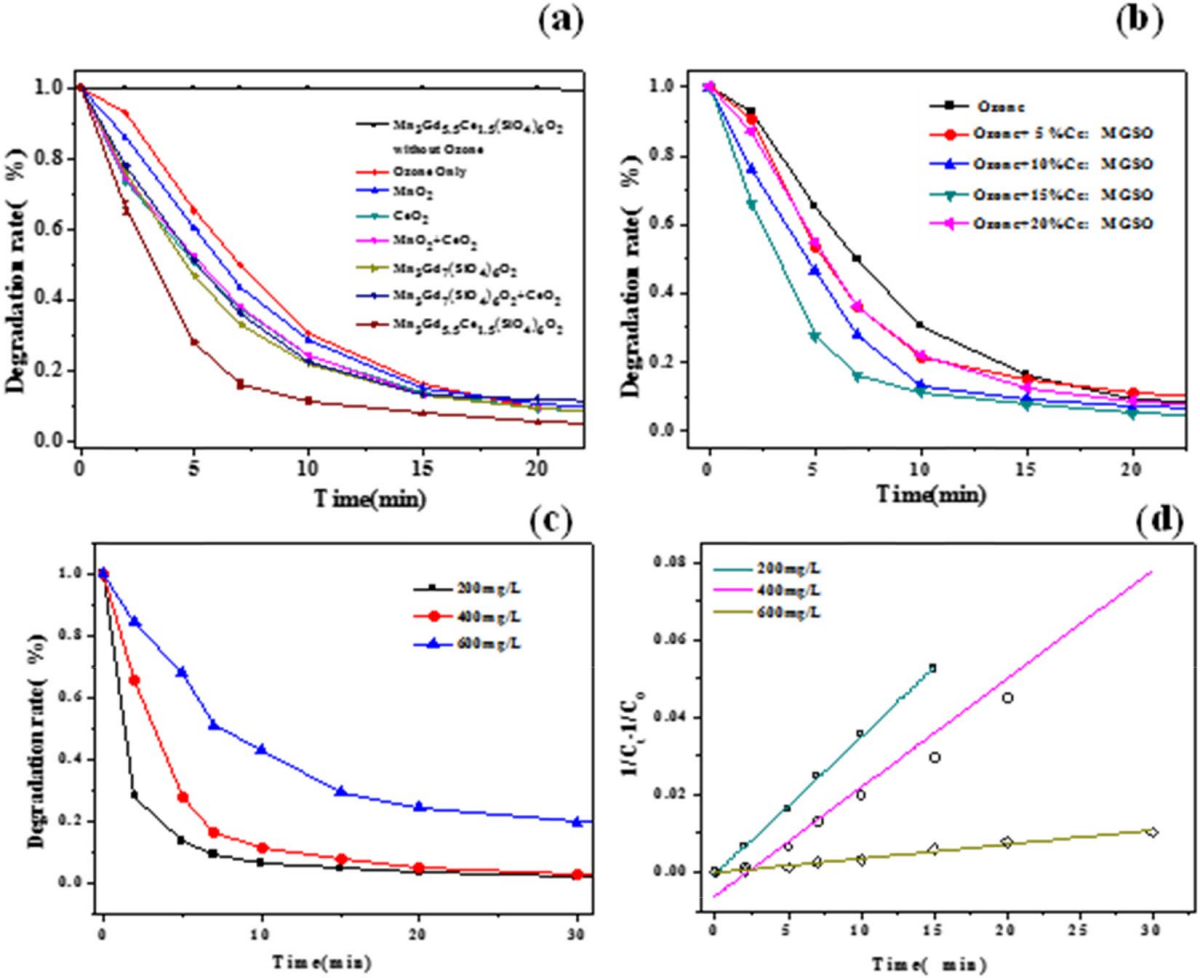
(**a**) XPS spectra of Mn_3_Gd_7−*x*_Ce_*x*_(SiO_4_)_6_O_1.5_ synthesized in present work. (**b**) high resolution XPS spectra at O 1 s position of compounds. (**c**) Mn 2p. position of Mn_3_Gd_7−*x*_Ce_*x*_(SiO_4_)_6_O_1.5_ (**d**) Ce 3d. position of Mn_3_Gd_7−*x*_Ce_*x*_(SiO_4_)_6_O_1.5_; (**e**) Comparative EPR spectra of Mn_3_Gd_7_(SiO_4_)_6_O_1.5_ and Mn_3_Gd_5.5_Ce_1.5_(SiO_4_)_6_O_1.5_ particles.

To investigate the chemical states of the variable elements and oxygen in the sample, a wide survey scan of XPS spectra was carried out. All peaks have been corrected by C1s peaks position (284.8 eV)^26^. Figure 4(a) shows the XPS survey spectrum of Mn_3_Gd_7–*x*_Ce_*x*_(SiO_4_)_6_O_1.5_. The presence of Mn, Gd, Ce, Si, O, and C in Fig. 4(a), with no other impurity was detected. The high resolution XPS scan of the Mn2p doublet with the peak deconvolution is shown in Fig. 4(c). Both the peaks of Mn could well attach to the Mn 2p3/2 and Mn 2p1/2 at the binding energies (BE) of 642.5 eV and 653.4 eV. There was no any noticeable shoulder peaks observed in Mn2p spectra of Mn_3_Gd_7−*x*_Ce_*x*_(SiO_4_)_6_O_1.5_, revealing that Mn ions are in the formal chemical valance state of 2+. Analysis of the Ce 3d spectra showed that 903.5 eV(U_0_), 898.8 eV(V′), and 884.9 eV(V_0_)were ascribed to Ce^3+^ species while 907.5 eV(U) and 888.2 eV(V) were attributed to Ce^4+^ species (Fig. 4(d)). The surface concentration of Ce^4+^ can be determined by Ce^4+^ = Ce^4+^/(Ce^4+^ + Ce^3+^); Ce^3+^ = Ce^3+^/(Ce^4+^ + Ce^3+^), where Ce^3+^  = U_0_ + V′ + V_0_ and Ce^4+^  = U + V. Inherent challenges are present for Ce 3d XPS spectrum analysis because of the difficulty in deconvolution of individual peaks in Ce 3d3/2 and Ce 3d5/2 envelopes^27^. So the proportion of Ce^4+^ and Ce^3+^ was 55% and 45% in the composite^28^. In order to confirm the oxygen vacancies in the prepared samples, we analyzed the XPS spectra of O1s. The result of O1s BE peaks of Mn_3_Gd_5.5_Ce_1.5_(SiO_4_)_6_O_1.5_ have been showed in Fig. 4(b). The O 1s XPS spectra were deconvoluted with three major peaks, located at 529.9 eV, 531.8 eV, and 532.6 eV, which correspond to lattice oxygen species (O_2_^−^) named O_I_ and adsorbed oxygen (e.g., O_2_^2−^ and O^−^), and hydroxyl groups (OH^−^), respectively^29^ named O_II_. The atom ratio of O_I_ to O_II_ was 4.02 for Mn_3_Gd_5.5_Ce_1.5_(SiO_4_)_6_O_1.5_, which is higher than that of Mn_3_Gd_7_(SiO_4_)_6_O_1.5_ to 3.17, respectively, indicating that the doping of Ce increased the oxygen defects of Mn_3_Gd_7_(SiO_4_)_6_O_1.5,_ which is the most active oxygen, and has been reported to play an important role in the oxidation reaction^30^. Otherwise, the EPR comparative experiment of Mn_3_Gd_7_(SiO_4_)_6_O_1.5_ and Mn_3_Gd_5.5_Ce_1.5_(SiO_4_)_6_O_1.5_ is shown in Fig. 4(e). Obviously, the peaks of Mn_3_Gd_5.5_Ce_1.5_(SiO_4_)_6_O_1.5_ is higher than that of Mn_3_Gd_7_(SiO_4_)_6_O_1.5,_ also verifing that the doping of Ce increases the oxygen vacancies, increases the activity of the catalyst.

### Catalytic degradation of TC in Mn_3_Gd_7−*x*_Ce_*x*_(SiO_4_)_6_O_1.5_ system

**Figure 5:**
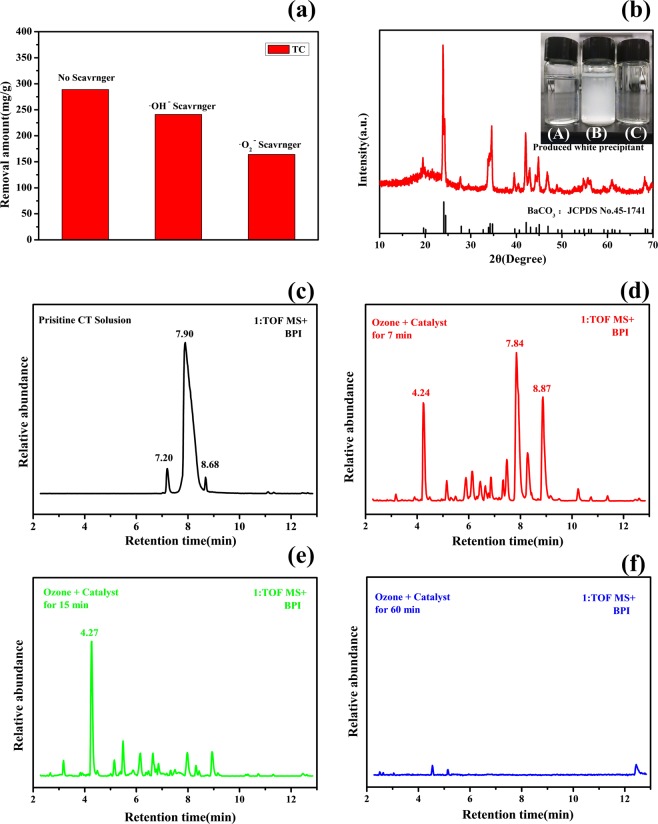
The catalytic catalystic degradation behavior of TC (Experimental conditions: Catalyst loading 2.5 g, pH = 3.4), [TC]_0_ = 400 mg L^−1^, working volume = 200 mL, and its degradation behavior under O_3_/Catalyst system, the concentration of ozone is 40 mg/L.) (**a**)different forms of manganese and cerium (**b**) different doping amount of cerium in the catalytic system (**c**)different initial concentrations in catalystic system (**d**) Pseudo-second order kinetic behavior under different tetracycline concentration in degradation process.

Degradation processes of TC using Mn_3_Gd_7−*x*_Ce_*x*_(SiO_4_)_6_O_1.5_ as a catalyst were investigated in the presence of ozone, (Experimental conditions: Catalyst loading 2 g L^−1^, pH = 3.4 working volume of 200 mL, [TC]_0_ = 400 mg L^−1^, the concentration of ozone is 40 mg/L). Figure 5(a) shows the comparisons of time-dependent reaction yields between the ozonation and the six catalysts (the situation under which ozone alone as a control is also included). First of all, when only the Mn_3_Gd_5.5_Ce_1.5_(SiO_4_)_6_O_1.5_ compound was added into the TC solution, there was no adsorption of TC. It is obvious that there are substantially different performances in the catalytic oxidation of TC. The composite Mn_3_Gd_5.5_Ce_1.5_(SiO_4_)_6_O_1.5_ has the greater catalytic capability for TC degradation in comparison with pure Mn_3_Gd_7_(SiO_4_)_6_O_1.5_. So, Mn_3_Gd_7_(SiO_4_)_6_O_1.5_ was mixed with CeO_2_, and MnO_2_. It could be observed, that the introduction of Ce enhanced the catalytic degradation efficiency. To further investigate the impact of Ce, different amounts of Ce doped Mn_3_Gd_7–*x*_Ce_*x*_(SiO_4_)_6_O_1.5_ samples was prepared, and used in the catalytic degradation of TC. As shown in Fig. 5(b), increasing of the Ce doping concentration from 0% to 15% led to a significant increase of TC removal, implying that the cerium doping plays an important role in catalytic ozonation as an active species. When the amount of cerium doping continuously increased up to 20%, the efficiency reduces. That can be explain that when the doping amount is 15%, (211) crystal plane of the compound has the lowest crystal strength, resulting in increased defects and active sites. So the most suitable proportion of Ce doped amount in Mn_3_Gd_7–*x*_Ce_*x*_(SiO_4_)_6_O_1.5_ is *x = *1.5 for catalytic ozonation of TC.

For ozonation catalyst process, initial TC concentration is a worth considering parameter. The experiments of different TC concentration were conducted, the result in Fig. 5(c) shows that the degradation efficiency of TC dropped with increasing of initial concentrations. When the concentration rises from 200 mg/L to 600 mg/L, the degradation efficiency dropped from 99.8% to 86.2%. The excess of TC (up to 600 mg/L) or its degradation intermediates may need to consume more active radicals, so the catalytic capability became slight low. Additionally, the mechanism of TC degradation under the catalysis of Mn_3_Gd_5.5_Ce_1.5_(SiO_4_)_6_O_1.5_ was explored under different initial TC concentration (200, 400 and 600 mg/L) according to the pseudo-first order and pseudo-second order kinetics^31^.

1 $${\rm{Pseudo}}-{\rm{first}}\,{\rm{order}}\,{\rm{kinetic}}:\,\mathrm{ln}\,({C}_{0}/{C}_{t})={k}_{1}{\rm{t}}$$

2 $${\rm{Pseudo}}-{\rm{second}}\,{\rm{order}}\,{\rm{kinetic}}:\,1/{C}_{t}-1/{C}_{0}={k}_{2}{\rm{t}}$$

where *C*_0_ is the initial concentration of TC, *C*_*t*_ is the concentration of the TC in the solution after treatment at time *t*, and *k*_1_ and *k*_2_ are pseudo-first and pseudo-second order rate constants. Clearly, the degradation of TC was better described by the pseudo-second order kinetic model^17^ judged by its regression coefficient (*R*^2^ > 0.96). The fitting result are shown in Fig. 5(d), as the concentration increased from 200 to 600 mg/L the reaction rate constants *k*_2_ were 0.0036, 0.0028 and 0.0004 Lmg^–1^ min^–1^, respectively.

### Possible degradation mechanism in the presence of Mn_3_Gd_5.5_Ce_1.5_(SiO_4_)_6_O_1.5_ catalyst

**Figure 6:**
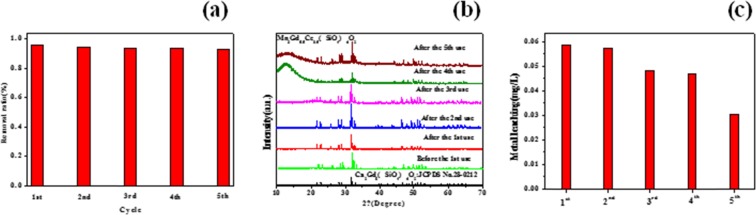
(**a**) The effects of different scavengers on the degradation of TC and with Mn_3_Gd_5.5_Ce_1.5_(SiO_4_)_6_O_1.5_ catalyst under O_3_, (**b**) XRD patterns of the white precipitation formed in Ba(OH)_2_ solutions, Insert: picture of Ba(OH)_2_ solutions after treatment. Gas produced by TC degradation with O_3_ (A) and gas produced by catalytic degradation passed through the Ba(OH)_2_ solutions.(B), several 2 mol/L HCl was added(C), High-performance liquid chromatography of TC (**c**), the degradation process for 7 min(**d**), 15 min(**e**), and 60 min(**f**).

During TC degradation with catalysts under ozone as oxidant, several kinds of reactive radicals, such as ·OH, and·O_3_, could be generated and have a great influence under the activation of transition metals. In order to investigate the effect of the two reactive radicals, benzoquinone and IPA were added into the TC solutions to scavenge these radicals. Benzoquinone is widely used to quench O_2_^−^, and IPA usually used to scavenge ·OH. After 5 min of reaction, 1 mmol IPA was added to the solution, the amount of TC degradation declined from 289 mg/L to 241 mg/L (Fig. 6(a)). When the same excess amount of benzoquinone was added, the TC removal dropped to 164 mg/L. The competitive radical tests suggest superoxide is the dominating active radicals in the degradation of TC using Mn_3_Gd_5.5_Ce_1.5_(SiO_4_)_6_O_1.5_ as a catalyst. Nevertheless, the results of the scavenger experiments proved that ·OH also participated in the catalytic ozonation, the combination of the two reactive radicals leads to the efficient reaction.

To confirm the catalytic effect of the Ce^3+^-doped composite, a couple of comparable experiments have been made. The catalytic degradation of TC using Mn_3_Gd_5.5_Ce_1.5_(SiO_4_)_6_O_1.5_ had a great efficiency than any others (Fig. 3(b)). Obviously, Ce made the difference in the process, as the XPS test showed Ce was made by a mixture of positive tri- and tetra-valent cations. Ce^3+^/Ce^4+^ is a good indicator for the redox reaction and development of superoxide free radicals^32^.

According to the above result, degradation reaction should occur on the catalyst surface via Ce due to its redox capability^11^. First, S ≡ Ce^3+^ could react with O_3_, with the electron transferring, superoxide and oxygen were produced^32,33^ (Eq. 3). Then the obtained O_2_^–^ continues to react with another O_3_ to produce a peroxide (O_2_^2–^) molecule and a dioxygen molecule (Eq. 4). O_2_^2–^ molecule would react with S ≡ Ce^4+^ in turn to produce Ce^3+^ (Eq. 5). The oxygen-containing reactive intermediate reacts with tetracycline which is also adsorbed on the surface of the catalyst^33^.

3 $${{\rm{O}}}_{3}+{\rm{S}}\equiv {{\rm{Ce}}}^{3+}+\cdot {\rm{OH}}\to {\rm{S}}\equiv {{\rm{Ce}}}^{4+}+{{\rm{O}}}_{2}+{{\rm{O}}}^{2-}$$

4 $${{\rm{O}}}_{3}+{{\rm{O}}}^{2-}\to {{\rm{O}}}_{2}+{{{\rm{O}}}_{2}}^{2-}$$

5 $${{{\rm{O}}}_{2}}^{2-}+{\rm{S}}\equiv {{\rm{Ce}}}^{4+}\to {{\rm{O}}}_{2}+{\rm{S}}\equiv {{\rm{Ce}}}^{3+}$$

As reported by Jia *et al*.^34^, the react active site would be made up of the oxygen vacancy site on the catalyst surface, when catalyst was attacked by ozone, one of O atom of O_3_ could insert into oxygen vacancy site. Electrons transfer will easy occur in oxygen vacancies to transfer electrons to an ozone molecule, the result is obtaining a new surface bound oxygen species (O^2−^) and dioxygen molecule at original oxygen vacancy site, which leads to be a gas phase (Eq. 6). The following step is that another ozone molecule will react with the surface bounded O^2−^ to form a dioxygen and the second peroxide (O_2_^2-^) molecule (Eq. 7). Finally, the peroxide species was unstable and decomposes to a dioxygen and then recover the initial oxygen vacancy to join the following ozonolysis cycle^33^. The key point of catalysis process is to decompose the peroxide in time to make sure the oxygen vacancies recover that the peroxide can easy transfer the lattice oxygen to improve the catalyst activity^33,34^.

6 $${{\rm{O}}}_{3}+{{\rm{V}}}_{{\rm{o}}}\to {{\rm{O}}}_{2}+{{\rm{O}}}^{2-}$$

7 $${{\rm{O}}}^{2-}+{{\rm{O}}}_{3}\to {{\rm{O}}}_{2}+{{{\rm{O}}}_{2}}^{2-}$$

8 $${{\rm{O}}}_{2}^{2-}\to {{\rm{O}}}_{2}+{{\rm{V}}}_{{\rm{o}}}$$

In order to further investigate the degradation process and intermediate compounds produced during reaction the HPLC-MS technology was applied. The results of the TC solution (400 mg/L) chromatography after different reaction time with the presence of the catalyst are illustrated in Fig. 6. The identification was based on mass fragmentation values and by comparing the mass spectra to a database. It is apparent, that the relative intensity of the ion [1 + H]^+^ of m/z 445 decreases with the reaction proceeds, whereas two new and intense ions with 461 m/z and 477 m/z are clearly detected. This means that the degradation reaction of TC solution continuously occurred. As the reaction proceeds three kinds of functional groups (double bond, amine group, and phenolic groups) of TC will compete for the ozone.

The results of the HPLC-MS indicate that fragmentations of TC yielded ions with an m/z value of 427 on the loss of NO_3_^−^, which further fragmented to the ions with the value 410 m/z on the H_2_O loss^35^. After 60 minutes of ozone catalytic degradation, there was still a small amount of compounds present (molecules with 114 m/z value were detected) (Fig. 6(d)).

However, the exact structures of these compounds could not be identified in the present study and further work still required for a more detailed structures analysis. In addition to these results, we also detected the exhausted gas generated during the degradation process, and the exhaust gas was introduced into the clarified saturated Ba(OH)_2_ solution as shown in the insert (A) in Fig. 6(b). It can be clearly observed that the transparent solution inset (A) Fig. 6(b) became cloudy inset (B) Fig. 6(b), and a certain amount of white precipitate was formed at the bottom of the bottle. When the excess dilute hydrochloric acid was added dropwise to the turbid solution, the cloudy disappeared and the solution became transparent again as shown in the insert (A) in Fig. 6(c). To identify the gained from the experience precipitate, it was collected from the bottom of the bottle and subjected to the XRD test. The XRD results are shown in the Fig. 6(b). The comparison with the card JCPDS no. 45–174 (Ba(CO_3_)_2_) revealed that the obtained precipitate is exactly Ba(CO_3_)_2_. This result confirmed that the reaction to produce CO_2_ gas as the final product of the degradation reaction progress of the TC took place in the experiment. The above experimental phenomena follows the following two reaction equations:

9 $${{\rm{CO}}}_{2}+{{\rm{Ba}}({\rm{OH}})}_{2}={{\rm{BaCO}}}_{3}\downarrow +{{\rm{H}}}_{2}{\rm{O}}$$

10 $${{\rm{BaCO}}}_{3}+{\rm{HCl}}={{\rm{BaCl}}}_{2}+{{\rm{H}}}_{2}{\rm{O}}+{{\rm{CO}}}_{2}\uparrow $$

The degradation process proceeds through the formation of a series of mineralized intermediates, which then further oxidize to water, inorganic ions and carbon dioxide^36^. Based on these results. So, it can be concluded that TC is completely decomposed through the oxidation by (•OH) and (•O^2−^) after less than 30 min the presence of ozone. The overall degraded reaction can be supposed by the following equation:

11 $${{\rm{C}}}_{22}{{\rm{H}}}_{24}{{\rm{N}}}_{2}{{\rm{O}}}_{8}+{{\rm{O}}}_{3}\to {{\rm{CO}}}_{2}+{{\rm{NO}}}_{3}^{-}+{{\rm{Cl}}}^{-}+{{\rm{H}}}_{2}{\rm{O}}$$

### Reusability and stability of Mn_3_Gd_5.5_Ce_1.5_(SiO_4_)_6_O_1.5_ catalyst

For economic reasons, the capability of recycling Mn_3_Gd_5.5_Ce_1.5_(SiO_4_)_6_O_1.5_ catalyst was evaluated. Figure 2(a) shows the results of catalyst after being used for five times (After each experiment, we used 10,000 rpm high speed centrifugation, and filtered the samples, then collected the samples, and dried at 80 °C in an air oven). Comparing with the first use, the degradation rate decreased slightly during the first and the fifth reactions, but TC removal was almost the same. This indicates that the catalyst still remains active after consecutive runs. XRD results shows that there is no impurity in the structure after each reaction, so the catalyst can keep a great reusability (Fig. 2(b)).

The amount of metal leaching is also an important factor in measuring the stability of the catalyst. The excessive metal leaching can lead to deactivation of the catalyst. So, an ICP measurement was carried out to determine the concentrations of dissolved active Ce after the reaction (Fig. 2(c)). When the amount of the added catalyst was 0.5 g per 200 mg/L TC solution at pH 3.4, the equilibrium concentrations of Mn, Gd, and Ce were 0.051, 0.050, and 0.086 mg/L, respectively, after the reaction was completed for 60 minutes. The amount of leached metal did not affect the activity of the catalyst, since the atoms are stable present in the apatite structure. So, a stable apatite structure can be applied as a potential structural design unit to heterogeneously catalyzed oxidative degradation.

In recent years, there are many kinds of ways have been intensively studied to completely settle an issue of tetracycline pollution in water. A preliminary and brief comparison of the degradation effects of different treating methods^37–41^ to remove tetracycline is summarized in Table 1. Compared with the mentioned works, the *as*-synthesized Mn_3_Gd_5.5_Ce_1.5_(SiO_4_)_6_O_1.5_ catalyst investigated in this study shows excellent performance for the degradation of tetracycline under ozone, and also shows outstanding reusability and stability.

**Table 1:** Comparison of the degradation effects of different treating methods to remove tetracycline.

method	Reaction conditions	Degradation	ref.
Mn_3_Gd_7−*x*_Ce_x_(SiO_4_)_6_O_2_/O_3_	[TC] = 400 mg/L [Catalyst] = 2.5 g/L	95% 20 min 99% 60 min	This work
Adsorption using mesoporous BiOI microspheres	[TC] = 40 mg/L	56% 360 min	^25^
US/Fe_3_O_4_/H_2_O_2_	[TC] = 100 mg/L [Catalyst] = 2.0 g/L	93.7% 60 min	^26^
Photocatalytic	[TC] = 40 mg/L [Catalyst] = 1.0 mg/L	95% 60 min	^27^
Microwave induction	[TC] = 50 mg/L [Catalyst] = 2.0 g/L	34.8% 30 min	^28^

## Conclusion

In this paper, the Mn_3_Gd_5.5_Ce_1.5_(SiO_4_)_6_O_1.5_ catalyst with the apatite-type structure was successfully prepared by traditional high temperature solid phase method. The catalyst exhibited good catalytic activity and stability for the degradation of TC at room temperature. This result could attribute to the synergistic effect between the different valence of the cerium ion. Free radical scavenging experiments proved that superoxide radicals were the most active substances in the reaction process, and, thus, suggested possible degradation pathways of TC in the reaction process. In summary, Mn_3_Gd_5.5_Ce_1.5_(SiO_4_)_6_O_1.5_ is a promising catalyst for removal of TC in wastewater.
